# Cross-border acquisition or greenfield investment? The role of investor sentiment

**DOI:** 10.3389/fpsyg.2022.1085286

**Published:** 2023-01-09

**Authors:** Li Dong, Jinlong Chen

**Affiliations:** Department of Business Administration, Huaqiao University, Quanzhou, China

**Keywords:** investor sentiment, OFDI, establishment mode, market timing effect, catering effect

## Abstract

When a firm invests in a foreign market, it has to choose between cross-border acquisition and greenfield investment. The impact of investor sentiment on the establishment mode of firm outward foreign direct investment (OFDI) has not been investigated by previous literature. Based on the data of Chinese listed companies between 2007 and 2019, this paper finds that firms prefer cross-border acquisition over greenfield investment when investor sentiment is high, and equity issuance and catering to sentiment are transmission channels. Cross-sectional test results show that the impact of investor sentiment on the establishment mode is concentrated in non-state-owned enterprises, and is stronger in non-eastern province firms and in technology-intensive OFDI. After various robustness tests, the main conclusion remains unchanged. This paper not only finds a new determinant that affects the establishment mode of OFDI but also enriches the research on the economic consequences of investor sentiment, which helps understand the role of investor sentiment in firms’ internationalization decisions.

## 1. Introduction

When a firm invests in a foreign market, it has to choose between cross-border acquisition and greenfield investment. Cross-border acquisition and greenfield investment are very different in terms of investment cost, time consumption, and flexibility. The acquisition allows firms to enter a foreign market and acquire foreign assets quickly; however, the cost for acquisition is usually higher than that for greenfield investment (Sun et al., 2021). In addition, the benefit of acquisition on the host country’s economic growth is limited because acquisition rarely involves physical investment such as plant, machinery, and equipment and does not help improve the host country’s employment rate. On the contrary, greenfield investment has to be established from scratch, which provides opportunities for economic growth and employment in the host country. Therefore, the choice of establishment mode is related to the firm and brings differentiated economic consequences to the host country.

The previous literature has conducted a panoply of investigations and research on the determinants of establishment mode. These determinants could be divided into three levels. The first is the country level, such as the market size of the host country (Al-Kaabi et al., 2010), the institutional environment (Brouthers and Brouthers, 2000; Meyer et al., 2009; Chen et al., 2017), and the economic policy uncertainty (Sun et al., 2021; Zhou et al., 2021). The second is the industry level, mainly the influence of industry heterogeneity (Hennart and Park, 1993; Anand and Delios, 2002; Larimo, 2003). Finally, there are firm-level factors, such as family ownership (Boellis et al., 2016; Yamanoi and Asaba, 2018; Rienda et al., 2019) and international strategy (Harzing, 2002). However, as far as we know, only a few works of literature have predicted the choice of establishment mode from the perspective of behavioral finance. Although Yamanoi and Asaba (2018) explained the impact of family ownership on the establishment mode from the socio-emotional wealth and risk aversion perspective, there needs to be literature to study from the perspective of investor irrationality. Investment decisions of listed companies are closely related to the stock market’s performance, and investors generally lack enough rationality. Driven by investor sentiment, stocks are commonly mispriced, which significantly impacts the decisions of acquisitions and other investment activities (Shleifer and Vishny, 2003; Polk and Sapienza, 2009; Shen et al., 2021). Unfortunately, investor sentiment has not attracted much attention in international business literature. Baker et al. (2008) are among the few scholars who introduced stock mispricing (a proxy for investor sentiment) into international business literature. They found that FDI flow is positively associated with the level of stock valuation of the source country and that the cheap financial capital hypothesis can explain this phenomenon. In the era of economic globalization, the performance of a country’s stock market may affect the flow and reallocation of its capital in the global scope, and the firm-level OFDI is the main carrier of cross-border capital flow. Therefore, it is significant to understand the impact of investor sentiment on firms’ OFDI decisions, which will help us understand the role of investor sentiment in cross-border capital flow and help the government formulate and improve financial regulatory policies. After Baker et al. (2008), we further consider the impact of investor sentiment (with stock mispricing as the proxy) on the establishment mode of OFDI.

China provides a suitable setting for our research. China has been one of the significant contributors to the growth of OFDI worldwide over the past two decades. Especially after the 2008 financial crisis, China’s OFDI has been accelerating, with an average annual growth rate of about 30%. In 2019, China’s total OFDI flow reached 136.91 billion dollars, ranking second in the world, while its total OFDI stock reached 2.2 trillion dollars, ranking third in the world. Therefore, Chinese firm-level OFDI and how Chinese firms choose the OFDI establishment mode will significantly impact the global economy. In addition, the preference of Chinese firms for the establishment mode of OFDI has changed significantly during this period, and the frequency and value of cross-border acquisitions are catching up to greenfield investments (Hu et al., 2022). The significant changes in the preference for the establishment mode help us observe investor sentiment’s impact on the establishment mode. Although the research object of this paper is listed companies and the number of listed companies is relatively small, the scale of assets, operating income, and profit contribution of listed companies play a significant role in China’s economy. At the end of 2021, there are 4,682 listed companies in China’s stock market, with a total market value of 96.53 trillion RMB; the annual total operating income of non-financial listed companies was nearly 54.9 trillion RMB in 2021, accounting for about 48% of China’s GDP. In addition, although most Chinese firms conducting OFDI are small and medium-sized firms, accounting for more than 80% of the total number, their OFDI is less than 20% of the total value (Zhou et al., 2021). Therefore, listed companies, leading firms in various industries, are the main force in conducting OFDI.

We apply the market timing and catering theories to analyze the relationship between investor sentiment and the establishment mode. Then, we use the data of Chinese listed companies from 2007 to 2019 to make an empirical examination. The relationship between investor sentiment and the establishment mode has a reasonable internal logic. First, managers will issue more shares to reduce the cost of capital when investor sentiment is high because the overpriced stock reduces the cost of equity financing. Cross-border acquisitions usually require more capital than greenfield investments; thus, the overpriced stock provides firms with cheaper capital for cross-border acquisitions. Second, cross-border acquisitions usually provide multinational firms, particularly those in emerging markets, with more long-term growth opportunities and higher short-term market performance than greenfield investment; thus, rational managers will cater to investor sentiment to maintain and improve the firm’s short-term market performance. The empirical results show that high investor sentiment will increase the propensity toward acquisition when firms choose the establishment mode of OFDI. Further evidence shows that high investor sentiment has increased equity issuance and provided adequate financial capital support for OFDI, which is conducive to firms overcoming the barrier of the high cost of cross-border acquisition so that they can quickly enter the host country market through acquisitions. The impact of investor sentiment on the establishment mode of OFDI is still significant in firms that do not have equity refinancing and firms with low equity dependence, indicating that the choice of establishment mode is also related to the catering effect. Finally, we find that the impact of investor sentiment on the establishment mode is concentrated in non-state-owned enterprises (non-SOEs), and is stronger in non-eastern province firms and in technology-intensive OFDI. We also conduct a series of robustness tests, including controlling for more variables, using an instrumental variable, replacing the proxy of investor sentiment, and excluding the potential influence of the “learning effect.” After these additional tests, the main conclusion is unchanged.

We make at least four contributions to the literature. First, this is the first paper to analyze the impact of investor sentiment on the establishment mode of OFDI. Our evidence supports the prediction of the cheap financial capital hypothesis (Baker et al., 2008) and further proposes and verifies a catering channel. The catering channel is a meaningful finding because it points out the influence of managers’ self-interest motivations on firms’ OFDI decisions under the impact of irrationalities of investors, and it is a supplement to the determinants of the OFDI decision. Additional cross-sectional tests also enrich our findings. Second, this paper enriches the literature on behavioral finance. The previous literature investigated the impact of investor sentiment on overall investment (Rhodes-Kropf et al., 2005; Dong et al., 2006; Grundy and Hui, 2010; Dong et al., 2012; Campello and Graham, 2013), R&D investment and innovation activity (Dang and Xu, 2018; Shen et al., 2021), cross-industrial capital flow (Hua et al., 2020), and corporate social responsibility (Naughton et al., 2019), but ignored the impact of investor sentiment on firms’ OFDI decisions, particularly on the choice of establishment mode. In the era of economic globalization, the belief or irrationality of investors in a country’s stock market may affect the economy of other countries, and OFDI is one of the essential bridges connecting the two; this paper has broadened our understanding of this aspect. This paper is also related to psychological literature. Behavioral finance is a cross-discipline of psychology and finance, and investor sentiment is one of the external manifestations of investor irrationality. There is much psychological literature on irrational human behavior and its economic consequences. This paper enriches the literature in the psychological area. Third, the investment behavior of firms in emerging economies is different from that in developed economies (Sun et al., 2021). Previous studies on OFDI and its establishment mode have focused on developed economies. As the largest emerging economy and the second largest source of FDI worldwide, China’s OFDI will profoundly impact the global economy. Therefore, taking China as the research object is beneficial to understanding firms’ OFDI behavior in emerging economies. In addition, China’s stock market is dominated by retail investors who are more irrational, which provides a good context for us to study the economic consequences of investor sentiment. Fourth, emerging economies such as China have different characteristics of economic development from developed countries, such as the proportion of SOEs in the economy, the imbalance of regional economic development, and the motivation of OFDI; we have enriched the research in those aspects through several cross-sectional tests. Exploring the role of state ownership is crucial in understanding the economic operation of emerging economies; we find that the impact of investor sentiment on the establishment mode of OFDI is mainly concentrated in non-SOEs, indicating that state ownership may be a boundary condition for investor sentiment’s impact on the establishment mode. In addition, we also examine the impact of firms’ locations and investment motivations on the relationship between investor sentiment and the establishment mode. In conclusion, our research is beneficial in understanding the internationalization behaviors of emerging-market firms.

The rest is organized as follows. Section 2 reviews the literature and develops hypotheses. Section 3 outlines the methodology and sample. Section 4 reports the empirical result. Section 5 is the cross-sectional analysis. Section 6 is the robustness test. Section 7 concludes the paper.

## 2. Literature review and hypothesis development

Investor sentiment has always been one of the focuses of behavioral finance literature. Behavioral finance theory originates from the query of the hypothesis of rational man and efficient market. Kahneman and Tversky (1979), psychologists, put forward the prospect theory, laying a solid foundation for the rise of behavioral finance theory. Kahneman et al. (1982) pointed out that some human behaviors are contrary to the basic hypothesis of the traditional investment decision-making model and are mainly reflected in three aspects: risk attitude, psychological account, and overconfidence. They called this phenomenon “cognitive bias.” Some scholars successively applied these psychological findings to finance studies (De Bondt and Thaler, 1985; Shefrin and Statman, 1994; Thaler, 1999), and much behavioral finance literature has emerged in the 21st century.

Behavioral finance theory argues that the market value does not always accurately reflect the actual value of the assets. Due to investor sentiment, asset prices will deviate from their actuals, affecting the firm’s financing and investment behavior. Scholars have proposed the market timing theory (Stein, 1996; Baker et al., 2003) and the catering theory (Polk and Sapienza, 2009). According to the market timing theory, a firm will choose to invest when the stock is overpriced because the overvaluation reduces the cost of equity capital so that the firm can provide cheap financial capital for investment activities by issuing low-cost shares. According to the catering theory, managers will rationally conduct catering investments to maintain the firm’s share price and maximize managers’ income.

Although Baker et al. (2008) empirically studied the relationship between investor sentiment and OFDI using the cross-border acquisition data of American multinational enterprises and found that investor sentiment has a positive impact on OFDI, their research also had some limitations. First, from their study, we can only understand the relationship between investor sentiment and cross-border acquisition; they ignored that greenfield investment is an essential part of OFDI. Second, they only analyzed “cheap financial capital channels” (market timing effect). It can be seen from other relevant literature that managers’ catering motivation may also be a significant reason for investor sentiment to affect the firm’s investment decision; however, they did not analyze the catering channel. Third, when making OFDI decisions, firms need to consider not only investment scale but also establishment mode. Fourth, as a developed country, the characteristics of the stock market and the motivations of OFDI in the United States are quite different from those in China. The conclusions drawn from their study may not apply to China and other similar emerging economies.

Based on extant literature, we study the impact of investor sentiment on the establishment mode of OFDI. We argue that investor sentiment may affect the establishment mode through equity issuance and catering channels.

### 2.1. Market timing effect (equity issuance channel)

Due to the significant difference in sunk costs between cross-border acquisition and greenfield investment (Nocke and Yeaple, 2007), a firm’s resource endowment will affect its choice of establishment mode. The cost of OFDI occurs in three stages. The first is the decision-making cost, that is, the cost of searching for information related to investment objectives, market sizes, laws, and institutions. The second is the direct cost: the firm must bear the cost of building plants and purchasing machinery and equipment when conducting a greenfield investment; it must pay the cost of purchasing the equity of the target foreign firm and the acquisition premium when conducting a cross-border acquisition. The last is the integration cost: the firm has to bear the cost of employee training, organizational culture construction, and establishing foreign sales networks.

In cross-border acquisition, the acquirer’s payments include the acquiree’s fundamental value and the acquisition premium (Pennings et al., 1994). In acquisitions, managers are often overly optimistic about the future return due to overconfidence, thus overvaluing the acquisition premium (Roll, 1986). Moreover, due to the more severe information asymmetry in cross-border acquisitions, acquirers cannot evaluate the value of the acquiree as they do in domestic acquisitions, which leads to a higher premium generated in cross-border acquisitions than in domestic acquisitions (Harris and Ravenscraft, 1991; Inkpen et al., 2000). The equity transfer requires firms to make a large one-time payment; on the contrary, firms can more flexibly and gradually put capital in the greenfield (Brouthers and Dikova, 2010). Therefore, the direct cost of acquisition will be higher than that of greenfield investment. In reality, cross-border acquisitions of Chinese firms usually involve a larger amount than greenfield investments. Statistics from the BVD (Zephyr) database show that more than 80% of cross-border acquisition cases of Chinese firms are completed through cash or one-time payment, which reflects the solid financial capability of Chinese cross-border acquirers. In contrast, greenfield investments require a smaller amount of capital. According to the Statistical Bulletin of China’s Foreign Direct Investment survey in 2017, the average amount of each cross-border acquisition case of Chinese firms reached 278 million dollars, while the average amount of each greenfield investment case was only about 46 million dollars. Obviously, firms that choose cross-border acquisition should have more financial resources.

For listed companies, optimistic investor sentiment is conducive to firms obtaining cheap financial capital from the stock market. They will also make investment decisions according to the movement of investor sentiment. Stein (1996) developed a theoretical model and elaborated the market timing theory. According to this theory, the emotional trading of irrational investors will cause the stock price to deviate from its actual value, and managers will issue (repurchase) more shares when the firm’s stock is overvalued (undervalued), which will affect the investment decisions of the firm. Baker et al. (2003) indicated that optimistic investor sentiment reduces the cost of equity capital and increases the scale of equity issuance, promoting investment. Rhodes-Kropf et al. (2005) decomposed firms’ market-book ratio (M/B) into fundamental value and mispricing components. They found that mispricing significantly impacts acquisition activities and that about 65% of acquisition activities come from 20% of the most overvalued acquirers. Campello and Graham (2013) found that when investor sentiment is high, firms’ financial constraints decrease, promoting investment. Baker et al. (2008) applied the stock valuation theory to OFDI and found that stock overvaluation provides cheap financial capital for firms and promotes OFDI.

Therefore, according to the market timing theory, since cross-border acquisitions require more financial resources than greenfield investments, firms will prefer cross-border acquisition over greenfield investment when investor sentiment is high.

### 2.2. Catering effect

The market timing theory assumes that firms must rely on external financing for new investment and does not consider the principal-agent issue. To maintain short-run stock prices and increase managers’ income, even when the firm has sufficient financial capital and does not rely on external financing, the managers will still invest more in projects with high risk but high returns to cater to high investor sentiment (Polk and Sapienza, 2009). For example, compared with physical investment, research and development (R&D) investment have a higher risk of failure; however, the competitiveness and profit of the firm can be rapidly improved once R&D is successful; firms will increase R&D investment to improve investors’ confidence in the firm’s future development (Bekkum et al., 2011). Shen et al. (2021) investigated the relationship between stock mispricing and R&D investment; they found that stock overpricing significantly promotes R&D investment and that this effect stems from equity issuance and catering effects. Cross-border acquisition usually faces more significant risks than greenfield investment, but may also provide more opportunities for firms’ future growth than greenfield investment. Therefore, investors may have higher expectations for cross-border acquisitions than for greenfield investments; the impact of investor sentiment on the establishment mode may also be related to catering effect. The detailed analysis is as follows.

Reuer and Ragozzino (2014) found that acquisition brought a series of risks related to adverse selection and moral hazard. As greenfield investment allows firms to put capital into an establishment gradually, it is more flexible and has lower risk than acquisition (Brouthers and Dikova, 2010). Firms entering the host country through greenfield investment can copy the existing institutional framework of the parent firm, transfer the knowledge and culture of the parent firm to the foreign subsidiary, and reduce the uncertainty related to the interaction with the foreign subsidiary (Hennart and Park, 1993; Barkema and Vermeulen, 1998; Boellis et al., 2016). Cross-border acquisitions also involve inherent risks that firms need much knowledge to manage cross-border acquisitions (Boermans and Roelfsema, 2013; Buckley et al., 2016c). Buckley et al. (2016a) believed it might be risky if a firm hopes to enter a host country quickly and efficiently by acquiring existing foreign firms. Due to the impact of multiple risks, cross-border acquisition has the issue of a low completion rate. For instance, according to statistics, the completion rate of cross-border acquisition of Chinese firms is only 67% after the 2008 financial crisis, which means that cross-border acquisitions conducted by Chinese firms have a failure probability of at least 30%. Boellis et al. (2016) found that cross-border acquisition has higher risk than greenfield investment and that the risk aversion of family ownership leads to the fact that multinational family firms tend to greenfield investment rather than cross-border acquisition.

Although cross-border acquisitions may not have many financial benefits in the short term (Gregory and McCorriston, 2005; Clougherty and Duso, 2009), they may bring more long-term growth opportunities for multinational firms, especially those in the emerging economy. By acquiring foreign firms, multinational firms can quickly acquire scarce resources, such as the knowledge base of foreign subsidiaries that are difficult to obtain through greenfield investment (Reiche et al., 2015). Emerging-market firms and investors see OFDI as a shortcut to acquiring strategic assets to improve competitiveness and catch up with competitors (Luo and Tung, 2007). These multinational firms broadly lack the resources and advantages needed to compete with local firms in the host country (Buckley et al., 2016b). Most literature believes that emerging-market multinationals are more willing to enter a foreign market through acquisition because acquisition is conducive to these multinational firms obtaining the strategic assets needed to compete with multinational firms in developed countries (Mathews, 2002; Luo and Tung, 2007; Gubbi et al., 2010). Acquirees, especially those in developed countries, can provide emerging-market multinationals with valuable assets, such as technology, brand, and sale network, for global competition (Buckley et al., 2016c; Haasis et al., 2018). By entering the host country through acquisition, multinational firms can better capture local business opportunities and even find potential resources that could increase the value of the multinational firm (Sirmon et al., 2007). Since OFDI can provide emerging-market firms with resources and capabilities they cannot acquire domestically, they must accelerate the internationalization process to achieve catch-up development (Mathews, 2002). Establishing subsidiaries from scratch takes more time than acquiring existing firms (Caves, 1982). Therefore, emerging-market multinationals could overcome the late-comer disadvantages and speed up the process of internationalization through cross-border acquisition. In a financial market with asymmetric information, investors often see firms’ high-risk financing and investment activities as a sign of high-risk-taking capacity; for example, firms show their risk-taking capacity to the outside by choosing risky debt maturity (Flannery, 1986). As cross-border acquisitions face higher risks and require more financial resources, firms can release signals through cross-border acquisitions to show investors their ambition and strength to participate in global competition (Gubbi et al., 2010; Tao et al., 2017). Moreover, achieving rapid expansion and growth is a strategy commonly adopted by firms in developing countries, which has also been encouraged and supported by the government (Chen and Shi, 2008). Cross-border acquisition promotes rapid overseas expansion of firms more than greenfield investment does. Many previous studies have shown that cross-border acquisition can improve the short-term market performance of firms, particularly firms in emerging markets, because investors usually have a positive and optimistic attitude toward the long-term growth opportunities that cross-border acquisition may bring to firms (Zhu and Malhotra, 2008; Nicholson and Salaber, 2013; Tao et al., 2017).

Therefore, high investor sentiment indicates that investors are confident in the future growth opportunities of the firm. In order to meet investors’ optimistic expectations for the firm and release positive signals to maintain and improve the firm’s share price, managers would like to choose an establishment mode with greater risk but faster entry speed, through which the firm can rapidly access the existing assets and knowledge of the host country. In particular, China’s multinational enterprises regard acquisition as the first choice to enter foreign markets (Peng, 2012). Therefore, we suppose that firms are more inclined to choose acquisition rather than greenfield investment due to the catering effect.

Consequently, we propose the following hypothesis:

*Hypothesis 1:* Due to the market timing effect and the catering effect, firms prefer cross-border acquisition over greenfield investment when investor sentiment is high.

## 3. Research design

### 3.1. Empirical model

To examine the impact of investor sentiment on the establishment mode, the following regression model is established:


(1) $$\begin{array}{c} OM_{i,j,t}=\alpha_{0}+\alpha_{1}SENT_{i,t}+\alpha_{i}\sum IC_{i,t-1}+\\ \;\;\;\;\;\;\;\;\;\;\alpha_{j}\sum JC_{j,t}+YEAR+IND+\varepsilon \end{array}$$

where *i*, *j*, and *t* represent the firm, country, and year, respectively. *OM* is a dummy variable indicating the establishment mode of OFDI, *SENT* is the investor sentiment, *IC* is a set of firm-level control variables, and *JC* is a set of country-level control variables. *YEAR* and *IND* represent time-fixed effect and industry-fixed effect, respectively. We lag the firm-level control variables by a year to alleviate the endogeneity. As our dependent variable is binary, we use the logit regression model for empirical analysis.

### 3.2. Measurement of variable

#### 3.2.1. OFDI establishment mode

When the foreign subsidiary is established through acquisition, *OM* equals 1; otherwise, *OM* equals 0.

#### 3.2.2. Investor sentiment

The previous literature shows that proxies for investor sentiment can be divided into market-level and firm-level sentiment. Baker and Wurgler (2006) used the principal component analysis method to construct a comprehensive index of market-level investor sentiment; however, this index ignores the cross-sectional difference in investor sentiment. When the market-level investor sentiment is high (low), the firm-level investor sentiment of partial firms may be low (high). Some literature uses stock mispricing to measure firm-level investor sentiment (Grundy and Hui, 2010; Xiang, 2022). We use stock mispricing as the proxy for firm-level investor sentiment. Following Rhodes-Kropf et al. (2005), we decompose the firm’s market-book ratio (M/B) to obtain the stock mispricing. The market-book ratio is decomposed as follows:


(2) ln(M/B)=m−b=(m−v)−(v−b)

In Equation 2, *m*, *v*, and *b* are the logarithms of the market value (*M*), fundamental value (*V*), and book value (*B*) of the firm, respectively. *m*–*v* is the stock mispricing.

We estimate the fundamental value of firms by regression model (3):


(3) $$\begin{array}{c} \ln{\left(\frac{M}{B}\right)}_{it}=\alpha_{oqt}+\alpha_{1qt}\ln{\left(\frac{M}{B}\right)}_{it}+\alpha_{2qt}\ln{\left(NI\right)}_{it}^{+}+\\ \;\;\;\;\;\;\;\;\;\;\alpha_{3qt}I_{\left(<0\right)}{\left(NI\right)}_{it}^{+}+\alpha_{4qt}LEV_{it}+\varepsilon_{it} \end{array}$$

where *NI* ^+^ is the absolute value of net income, and *LEV* is leverage. *I*_(<0)_ is the indicator function. When net income is negative, the value of *I*_(<0)_ is equal to 1; otherwise, it equals 0. We regress Equation 3 for each industry (*q*) and obtain the regression coefficients {*α*_0*qt*_, *α*_1*qt*_, *α*_2*qt*_, *α*_3*qt*_, *α*_4*qt*_}. We calculate the average value of the regression coefficients of each period in the same industry; then, the equation of each industry is obtained. We then put the financial data of each period of the firm into the equation to estimate each firm’s fundamental value (*V*) in each period. Finally, we calculate the stock’s mispricing (*MISP*):


(4) $$MISP_{it}=m_{it}-v_{it}$$

When *MISP* is greater (less) than 0, the firm is overvalued (undervalued). We use *MISP* as the proxy for investor sentiment. The bigger the *MISP*, the higher the investor sentiment. As investor sentiment in China frequently fluctuates, we use more frequent quarterly data for calculation and take the mean value of quarterly *MISP* in that year as the proxy for investor sentiment (*SENT*).

#### 3.2.3. Control variables

Firm size (*SIZE*) is measured by the logarithm of total assets. Small firms are more flexible in acquisition activities than large firms; thus, small multinational firms have higher acquisition performance and are thus more likely to choose acquisition than large firms (Wilson, 1980). However, because large firms have advantages in financial resources and management capabilities, they are more inclined to choose acquisition than small firms (Larimo, 2003).

Productivity (*TFP*) is measured by the total factor productivity calculated by the ACF method (Ackerberg et al., 2015). High-productivity firms usually have more advantageous organizational and management capabilities than low-productivity firms; thus, they have a stronger ability to integrate assets in the post-acquisition and have lower integration costs than low-productivity firms do. In addition, high-productivity firms’ cross-border acquisitions may have a positive technology spillover effect on host countries, promoting the technological progress of host countries; thus, high-productivity firms may face lower scrutiny costs from the host country. Therefore, high-productivity firms are more likely to choose acquisition than low-productivity firms.

Leverage (*LEV*) is measured by the asset-liability ratio. Leverage reflects the debt risk and debt financing capacity of firms. A high leverage ratio may constrain the capacity of mortgage loans of the firm but also may improve the capacity of OFDI due to the increase of external financial capital (Buch et al., 2014).

Capital intensity (*CI*) is measured by the logarithm of the ratio of net fixed assets to the number of employees. Compared with the integration of human resources, the integration of assets after an acquisition is less challenging (Hennart and Reddy, 1997). Capital-intensive firms mainly aim at the integration of foreign assets. Therefore, the greater the firm’s capital intensity, the less complicated the integration is, and the more likely it is to choose acquisition.

Management expense ratio (*ME*) is measured by the ratio of the management expense to the operating income. On the one hand, the management expense ratio reflects the principal-agent issue (Ang et al., 2000). Managers will increase personal income through acquisitions (Fu et al., 2013). On the other hand, the management expense ratio reflects the level of investment in organizational management. Managers may choose cross-border acquisition to increase personal incomes if the management expense ratio reflects the principal-agent issue. If the management expense ratio reflects the intensity of firms’ investment in organizational management that may strengthen firms’ ability in acquisition integration, firms will improve the possibility of acquisition.

Ownership (*SOE*). OFDI flow is related to state ownership (Huang et al., 2017), so state ownership may also impact the establishment mode. If the state controls the firm, *SOE* is equal to 1; otherwise, *SOE* is equal to 0.

The market size of the host country (*MS*), is measured by the *per capita* GDP of the host country. When the host country’s market is enormous, it is the strategic choice of most multinational firms to conduct a greenfield investment (Al-Kaabi et al., 2010).

The market growth of the host country (*MG*) is measured by the host country’s real GDP growth rate and reflects the host country’s market prospects and investment potential. On the one hand, in the slow-growth host country market, local firms face more fierce competition, and more local firms are willing to be acquired (Brouthers and Brouthers, 2000). On the other hand, if the host country market grows at high speed, firms will face higher opportunity costs if they enter through greenfield investment due to the slower entry speed (Hennart and Park, 1993).

Investment freedom of the host country (*IF*). In the acquisition process, the fixed assets investment is generally not increased and even accompanies layoffs. Therefore, the host country’s scrutiny of cross-border acquisition is often more prudent than that of greenfield investment. Moskalev (2010) investigated samples from 57 countries and found that in countries with higher investment freedom, the number of acquisitions increased more significantly than that of greenfield investments.

### 3.3. Sample and data

We obtained data on OFDI projects of Chinese non-financial listed companies between 2007 and 2019. Chinese listed companies disclose the information of their associated foreign firms in financial reports; the information is recorded in the CSMAR database. We define the OFDI project as the new foreign subsidiary establishment (shareholding ratio exceeds 10%) in a given year. Foreign subsidiaries located in tax havens such as Cayman, Panama, and Bermuda are excluded because firms registered in these places are usually for tax avoidance. In addition, we exclude foreign subsidiaries in Hong Kong and Macao because these projects involve a large amount of round-tripping that cannot truly reflect OFDI (Sutherland and Ning, 2011). We obtained 4,879 OFDI projects distributed in 100 countries from 1,027 Chinese non-financial listed companies.

The other financial data also come from the CSMAR database. The original country-level control variables are from the World Bank and are recorded in the CSMAR database. We match the independent variable with the dependent variable according to the code and year of the listed company and country. We winsorize the top and bottom 1% of firm-level continuous variables. Finally, 4,879 firm-year-country observations are obtained.

Tables 1 and 2 report the descriptive statistical result and the correlation matrix, respectively. The mean value of *OM* is 0.377, indicating that 37.7% of OFDI projects are established through acquisition, which is lower than the proportion of greenfield investment. The mean value of *SENT* is 0.156, and the standard deviation is 0.392, indicating that investor sentiment varies significantly between firms and different years and that the stock prices of firms that have made OFDI are generally overpriced. The other variables are within a reasonable range. The correlation between *OM* and *SENT* (0.068, *p <* 0.01) provides preliminary evidence for our hypothesis. Most correlation coefficients are below 0.5, and the VIF of each variable is below 5, indicating that there is no severe multicollinearity.

**Table 1:** Descriptive statistics.

	Mean	Median	SD	Min	Max	*N*
*OM*	0.377	0	0.485	0	1	4,879
*SENT*	0.156	0.131	0.392	−0.717	1.219	4,879
*SIZE*	22.78	22.67	1.343	20.240	26.72	4,879
*TFP*	11.570	11.49	0.781	10.010	13.74	4,879
*LEV*	0.461	0.457	0.196	0.056	0.866	4,879
*CI*	12.47	12.45	1.027	9.705	15.02	4,879
*ME*	0.095	0.084	0.063	0.011	0.369	4,879
*SOE*	0.242	0	0.429	0	1	4,879
*MS*	10.02	10.66	1.272	5.762	11.48	4,879
*MG*	2.649	2.331	2.149	−20.49	17.41	4,879
*IF*	0.687	0.750	0.193	0	0.900	4,879

**Table 2:** Correlation matrix.

	1	2	3	4	5	6	7	8	9	10	11	VIF
1.*OM*	1											
2.*SENT*	0.068^***^	1										1.09
3.*SIZE*	−0.026	0.108^***^	1									2.12
4.*TFP*	0.021	0.039	0.552^***^	1								1.78
5.*LEV*	−0.017	−0.020	0.582^***^	0.412^***^	1							1.64
6.*CI*	0.057^***^	−0.139^***^	0.292^***^	0.100^***^	0.253^***^	1						1.19
7.*ME*	0.010	0.134^***^	−0.396^***^	−0.533^***^	−0.424^***^	−0.250^***^	1					1.61
8.*SOE*	−0.010	−0.057^***^	0.396^***^	0.267^***^	0.272^***^	0.179^***^	−0.199^***^	1				1.22
9.*MS*	0.124^***^	0.057^***^	−0.053^**^	−0.082^***^	−0.094^***^	−0.001	0.119^***^	−0.087^***^	1			3.68
10.*MG*	−0.116^***^	−0.013	0.054^***^	0.045	0.050^**^	−0.022	−0.051^**^	0.044	−0.578^***^	1		1.51
11.*IF*	0.151^***^	0.047^*^	−0.045^*^	−0.079^***^	−0.096^***^	0.011	0.128^***^	−0.096^***^	0.818^***^	−0.442^***^	1	3.06

^***^p < 0.01; ^**^p < 0.05; ^*^p < 0.1.

## 4. Empirical results analysis

### 4.1. Univariate analysis

We conduct a univariate analysis to preliminarily examine the impact of investor sentiment on the establishment mode. The sample is divided into the pessimism group and the optimism group. When *SENT* is less (greater) than 0, it indicates that the firm is undervalued (overvalued) and that investor sentiment is pessimistic (optimistic). Table 3 reports the difference and significant level of *OM* between the two groups. The mean value of *OM* of the pessimistic group is less than that of the optimistic group, with a difference of 0.084 (*p* < 0.01). The result shows that when investors are optimistic, firms prefer cross-border acquisitions over greenfield investments.

**Table 3:** Univariate analysis.

Variable	Pessimistic sentiment group		Optimistic sentiment group		Difference
	*N*	Mean	*N*	Mean	*t*-Test
*OM*	1,687	0.322	3,192	0.406	−0.084^***^

^***^p < 0.01.

### 4.2. Baseline regression results

Table 4 reports the regression results of model (1). Column (1) only contains the core explanatory variable (*SENT*); Column (2) includes the firm-level control variables; Column (3) further includes country-level control variables; and Column (4) further controls for the year-fixed and industry-fixed effects. It can be seen that, from column (1) to column (4), along with other variables are controlled for, although the value of the coefficient of *SENT* has changed, it is always significantly positive (*p* < 0.01), indicating that high investor sentiment significantly increases the propensity toward cross-border acquisition (vs. greenfield investment).

**Table 4:** Investor sentiment and foreign establishment mode.

	(1)*OM*	(2)*OM*	(3)*OM*	(4)*OM*
*SENT*	0.358^***^	0.452^***^	0.447^***^	0.597^***^
	(0.074)	(0.078)	(0.080)	(0.102)
*SIZE*		−0.140^***^	−0.152^***^	−0.187^***^
		(0.033)	(0.034)	(0.040)
*TFP*		0.195^***^	0.202^***^	0.155^**^
		(0.050)	(0.051)	(0.065)
*LEV*		−0.086	0.015	0.470^**^
		(0.191)	(0.195)	(0.221)
*CI*		0.194^***^	0.177^***^	0.117^***^
		(0.031)	(0.032)	(0.041)
*ME*		0.733	0.231	2.102^***^
		(0.595)	(0.602)	(0.727)
*SOE*		0.002	0.067	0.061
		(0.078)	(0.080)	(0.090)
*MS*			−0.080^*^	−0.083
			(0.045)	(0.053)
*MG*			−0.074^***^	−0.081^***^
			(0.018)	(0.022)
*IF*			1.833^***^	1.696^***^
			(0.282)	(0.310)
*Constant*	−0.560^***^	−2.097^**^	−1.980^**^	1.057
	(0.032)	(0.821)	(0.906)	(1.293)
*YEAR*	No	No	No	Yes
*IND*	No	No	No	Yes
*N*	4,879	4,879	4,879	4,879
Pseudo *R*^2^	0.003	0.012	0.032	0.107

^***^p < 0.01; ^**^p < 0.05; ^*^p < 0.1. Standard errors in parentheses.

Regarding the control variables, the coefficient of *SIZE* is significantly negative, indicating that small firms prefer cross-border acquisition over greenfield investment. The reason may be that small firms have higher flexibility and performance in acquisition activities (Wilson, 1980). The coefficient of *TFP* is significantly positive, indicating that the higher the productivity of firms, the more inclined they are to acquisition; the reason may be that high-productivity firms usually have more advantageous organizational and management capabilities to integrate assets than low-productivity firms. In addition, the coefficients of *CI*, *ME*, and *IF* are significantly positive, indicating that the acquisition probability is positively associated with capital intensity, management expense, and investment freedom. The coefficient of *MG* is significantly negative, indicating that the probability of acquisition is negatively associated with the market growth of the host country.

Therefore, the baseline regression results support our hypothesis, and the regression coefficients of the control variables can also be reasonably explained.

### 4.3. Transmission channels analysis

Investor sentiment may impact the establishment mode through equity issuance and catering channels. To examine whether these two transmission channels are statistically significant, we follow Polk and Sapienza (2009) and add the variable of equity issuance (*EF*) to the regression model:


(5) $$\begin{array}{l} OM_{i,j,t}=\alpha_{0}+\alpha_{1}SENT_{i,t}+\alpha_{2}EF_{i,t}+\\ \;\;\;\;\;\;\;\;\;\;\;\;\;\;\;\;\;\;\alpha_{i}\sum IC_{i,t-1}+\alpha j\sum JC_{j,t}+\sum YEAR+\sum IND+\varepsilon \end{array}$$

where *EF* = the cash received from issuing shares/the total assets. The regression result is shown in column (1) of Table 5. The coefficient of *EF* is significantly positive, which directly verifies the equity issuance channel. After excluding the equity issuance channel, the coefficient of *SENT* is still significantly positive, which indirectly verifies the catering channel.

**Table 5:** Transmission channels analysis.

	(1)*OM*	(2)*OM*	(3)*OM*
*SENT*	0.504^***^	0.996^***^	0.488^**^
	(0.104)	(0.227)	(0.226)
*EF*	0.862^***^		
	(0.199)		
*SIZE*	−0.150^***^	−0.073	−0.225^***^
	(0.041)	(0.093)	(0.082)
*TFP*	0.178^***^	0.419^***^	0.301^**^
	(0.065)	(0.131)	(0.126)
*LEV*	0.327	0.937^*^	1.697^***^
	(0.226)	(0.501)	(0.487)
*CI*	0.118^***^	0.013	−0.083
	(0.041)	(0.080)	(0.084)
*ME*	1.991^***^	5.257^***^	4.812^***^
	(0.744)	(1.484)	(1.526)
*SOE*	0.037	0.272	0.302
	(0.090)	(0.196)	(0.187)
*MS*	−0.082	−0.266^**^	−0.073
	(0.053)	(0.108)	(0.109)
*MG*	−0.082^***^	−0.127^***^	−0.086^**^
	(0.022)	(0.046)	(0.043)
*IF*	1.652^***^	2.214^***^	1.416^**^
	(0.311)	(0.660)	(0.642)
*Constant*	0.026	−4.003	2.850
	(1.321)	(2.686)	(2.474)
*YEAR*	Yes	Yes	Yes
*IND*	Yes	Yes	Yes
*N*	4,879	1,185	1,312
Pseudo *R*^2^	0.110	0.124	0.159

^***^p < 0.01; ^**^p < 0.05; ^*^p < 0.1. Standard errors in parentheses.

The method proposed by Baker et al. (2003) is also used to examine the catering channel in this paper. If investor sentiment impacts the establishment mode through a catering channel, the positive effect of investor sentiment on the establishment mode will still be significant in firms with low equity dependence. We create the index of equity dependence (*ED*), *ED* = (the change in net assets−the change in retained earnings)/the total assets. If *ED* is below 0, the firm’s equity dependence is low. We only retain the firms with low equity dependence to re-examine the model (1). The result is shown in column (2) of Table 5. The coefficient of *SENT* is still significantly positive. So, there is a catering channel.

According to Polk and Sapienza (2009), if investor sentiment impacts firm investment through a catering channel, investor sentiment will also impact investment when the firm does not rely on external equity financing. Therefore, we refer to Polk and Sapienza (2009) and retain only the firms with no equity refinancing to re-examine the model (1). The regression result is shown in column (3) of Table 5. The coefficient of *SENT* is still significantly positive.

On the whole, both the equity issuance channel and the catering channel are proven by empirical evidence.

## 5. Cross-sectional analysis

### 5.1. State ownership

We investigate whether the impact of investor sentiment on the establishment mode is significantly different between SOEs and non-SOEs. Columns (1) and (2) of Table 6 report the regression results of the subsample of SOEs and non-SOEs, respectively. It can be seen that in the subsample of SOEs, the coefficient of *SENT* is insignificant. In the subsample of non-SOEs, the coefficient of *SENT* is significantly positive. Therefore, the effect of investor sentiment on the establishment mode is concentrated in non-SOEs.

**Table 6:** Cross-sectional analysis.

	SOEs	Non-SOEs	Eastern provinces	Non-eastern provinces	Technology-intensive OFDI	Non-technology-intensive OFDI
	(1)*OM*	(2)*OM*	(3)*OM*	(4)*OM*	(5)*OM*	(6)*OM*
*SENT*	0.176	0.798^***^	0.570^***^	0.726^***^	1.685^***^	0.509^***^
	(0.272)	(0.125)	(0.118)	(0.273)	(0.362)	(0.110)
*SIZE*	−0.404^***^	−0.076	−0.220^***^	−0.045	−0.427^***^	−0.155^***^
	(0.093)	(0.050)	(0.047)	(0.104)	(0.163)	(0.042)
*TFP*	0.354^**^	0.165^**^	0.105	0.274^*^	0.364	0.155^**^
	(0.170)	(0.075)	(0.079)	(0.144)	(0.239)	(0.070)
*LEV*	−0.014	0.396	0.280	1.679^***^	1.921^**^	0.213
	(0.536)	(0.263)	(0.258)	(0.559)	(0.820)	(0.240)
*CI*	0.008	0.084^*^	0.163^***^	−0.024	0.033	0.131^***^
	(0.106)	(0.050)	(0.049)	(0.103)	(0.138)	(0.045)
*ME*	5.745^*^	1.770^**^	1.719^*^	4.111^**^	−0.738	2.983^***^
	(3.349)	(0.791)	(0.878)	(1.746)	(2.102)	(0.834)
*SOE*			0.100	−0.010	0.695^*^	−0.002
			(0.109)	(0.212)	(0.395)	(0.094)
*MS*	0.304^***^	−0.180^***^	−0.007	−0.348^***^	−0.027	−0.067
	(0.099)	(0.062)	(0.064)	(0.111)	(0.204)	(0.056)
*MG*	0.025	−0.118^***^	−0.077^***^	−0.107^**^	−0.098	−0.078^***^
	(0.032)	(0.025)	(0.028)	(0.042)	(0.088)	(0.022)
*IF*	0.104	2.037^***^	1.556^***^	1.975^***^	2.035	1.630^***^
	(0.576)	(0.377)	(0.371)	(0.624)	(1.423)	(0.323)
*Constant*	4.521^*^	−2.007	2.519	15.531^***^	1.712	0.160
	(2.526)	(1.615)	(1.693)	(3.026)	(3.900)	(1.355)
*YEAR*	Yes	Yes	Yes	Yes	Yes	Yes
*IND*	Yes	Yes	Yes	Yes	Yes	Yes
*N*	1,183	3,696	3,753	1,126	508	4,340
Pseudo *R*^2^	0.193	0.133	0.126	0.202	0.174	0.114
Chow test *p*-value	0.007		0.011		0.000	

^***^p < 0.01; ^**^p < 0.05; ^*^p < 0.1. Standard errors in parentheses.

There are two possible reasons for this difference. First, state-owned banks account for much of China’s banking and have natural links with SOEs. Therefore, SOEs have advantages in obtaining loans from banks. Moreover, SOEs have an “implicit guarantee” provided by the government; non-SOEs face discrimination when applying for loans (Song et al., 2011). Therefore, non-SOEs will be more sensitive to changes in conditions of external equity financing when making OFDI decisions. Thus the market timing effect will be stronger for SOEs than for non-SOEs.

Second, different incentive mechanisms and business goals lead to differences in catering investment between SOEs and non-SOEs. For a long time, the Chinese government has regulated and restricted the remuneration of executives of SOEs. The higher the pay-performance sensitivity, the more consistent the share price and the managers’ interest motivation will be (Buck et al., 2008). The remuneration regulation of SOEs will reduce the impact of the stock market factors on the relationship between stock price and executive remuneration because no matter how high the stock price is, the improvement of executive remuneration is limited. Moreover, SOEs serve economic and political goals, but the main goals are political ones, such as providing employment and social welfare (Lin et al., 1998; Cuervo-Cazurra et al., 2014). Therefore, the managers of non-SOEs will pay more attention to the firm’s market value than those of SOEs do because the firm’s market value is a crucial indicator for assessing shareholders’ wealth and the managers’ performance.

### 5.2. Location

Considering the massive imbalance between the eastern and non-eastern provinces in terms of economy, population, and business environment, we investigate whether the impact of investor sentiment on the establishment mode of OFDI is associated with firms’ locations. The previous literature found the location’s influence on the relationship between firms’ external factors (e.g., economic policy uncertainty) and the choice of establishment mode (Zhou et al., 2021). Columns (3) and (4) of Table 6 report the regression results of subsamples of the eastern and non-eastern provinces, respectively. In the two groups, the coefficients of *SENT* are both significantly positive, but the coefficient of *SENT* of the non-eastern group is greater than that of the eastern group. We conduct the Chow test to examine the coefficient difference between the two groups, and the result shows that the coefficient difference of *SENT* between the two groups is significant (*p* < 0.05). Therefore, the impact of investor sentiment on the establishment mode of OFDI is more pronounced in non-eastern province firms than in eastern province firms.

This difference may be because, compared to non-eastern provinces, eastern provinces have developed economies and rich financial resources. Firms in the eastern provinces have advantages in obtaining financial resources; thus, they can carry out leveraged buyouts with the support of credit resources without having to wait until the stock is overvalued to acquire foreign firms.

### 5.3. The type of OFDI

We divide OFDI into technology-intensive and non-technology-intensive ones according to the investment motivation. Columns (5) and (6) represent the regression results of the technology-intensive OFDI group and the non-technology-intensive OFDI group, respectively. The coefficients of *SENT* of the two groups are both significantly positive. However, the coefficient of *SENT* of the technology-intensive group is greater than that of the non-technology-intensive group. We conduct the Chow test to examine the coefficient difference between the two groups, and the result shows that the coefficient difference of *SENT* between the two groups is significant (*p* < 0.01). Therefore, the impact of investor sentiment on the establishment mode is more pronounced in technology-intensive OFDI than in non-technology-intensive OFDI.

The reasons for this difference may be as follows. First, technology-intensive foreign subsidiaries have higher technology reserves and innovation potentials than non-technology-intensive foreign subsidiaries; thus, the premium of technology-intensive acquisition is higher than that of non-technology-intensive acquisition, leading to a higher requirement for the acquirer’s financial capacity. Therefore, the market timing effect will be stronger for technology-intensive OFDI than for non-technology-intensive OFDI. Second, acquiring foreign technology-intensive firms provide emerging-market multinationals with the advanced technology needed for global competition, which gains more favor with investors. Therefore, the catering effect will be stronger for technology-intensive OFDI than for non-technology-intensive OFDI.

## 6. Robustness tests

First, we replace the proxy of investor sentiment. Some literature obtained the irrational component in stock price and used it as the proxy for investor sentiment by decomposing Tobin’s Q (Goyal and Yamada, 2004; Hua et al., 2020). We refer to them and select the firm size, return on net assets, the growth rate of sales, and assets-to-liability ratio as the independent variables, while the dependent variable is Tobin’s Q. Then, the OLS model is regressed for each industry and period, where the fitted value reflects the firm’s actual value and the residual reflects the stock mispricing. The residual is taken as the new proxy for investor sentiment. Column (1) of Table 7 represents the regression result after replacing the proxy of investor sentiment. The coefficient of *SENT* is still significantly positive.

**Table 7:** Robustness tests.

	(1)*OM*	(2)*OM*	(3)*OM*	(4)*OM*
*SENT*	0.076^**^	0.645^***^	0.129^***^	0.524^***^
	(0.031)	(0.132)	(0.027)	(0.109)
*SIZE*	−0.136^***^	−0.304^***^	−0.039^***^	−0.197^***^
	(0.043)	(0.052)	(0.008)	(0.042)
*TFP*	0.219^***^	0.209^**^	0.031^**^	0.170^**^
	(0.069)	(0.082)	(0.013)	(0.069)
*LEV*	0.669^***^	0.490^*^	0.100^**^	0.564^**^
	(0.233)	(0.280)	(0.045)	(0.238)
*CI*	0.096^**^	0.214^***^	0.023^***^	0.066
	(0.043)	(0.053)	(0.008)	(0.043)
*ME*	2.719^***^	2.664^***^	0.433^***^	2.144^***^
	(0.751)	(0.986)	(0.154)	(0.766)
*SOE*	−0.087	0.082	0.012	0.101
	(0.094)	(0.115)	(0.018)	(0.096)
*MS*	−0.103^*^	−0.007	−0.015	−0.663
	(0.055)	(0.067)	(0.010)	(0.528)
*MG*	−0.074^***^	−0.074^**^	−0.015^***^	−0.016
	(0.022)	(0.029)	(0.004)	(0.037)
*IF*	1.740^***^	1.684^***^	0.325^***^	−2.639^***^
	(0.322)	(0.384)	(0.059)	(0.844)
*INC*				3.441^*^
				(1.878)
*ID*				−0.195
				(0.673)
*Constant*	−0.271	1.428	0.728^***^	8.430
	(1.332)	(1.648)	(0.267)	(5.387)
*YEAR*	Yes	Yes	Yes	Yes
*IND*	Yes	Yes	Yes	Yes
*COUN*	No	No	No	Yes
*N*	4,575	2,954	4,855	4,870
Pseudo *R*^2^	0.104	0.139	0.133	0.159

^***^p < 0.01; ^**^p < 0.05; ^*^p < 0.1. Standard errors in parentheses.

Second, we exclude the influence of the “learning effect.” We retain only the sample of the first OFDI of the firm, for that prior OFDI experience may affect the current OFDI establishment mode (Barkema and Vermeulen, 1998; Zhou et al., 2021). We select the samples of firms’ first OFDI toward a given host country and then use the selected samples to re-examine the model (1). Column (2) of Table 7 shows the result; the coefficient of *SENT* is still significantly positive.

Third, we use an instrumental variable to alleviate endogeneity. We follow Hua et al. (2020) and use lagged investor sentiment as the instrumental variable. Due to the dependent variable being a dummy variable, we use the linear probability model (LPM) for regression. The instrumental variable passed the under-identification test and the weak-identification test. Column (3) of Table 7 shows the regression result in the second stage of 2SLS; the coefficient of *SENT* is significantly positive.

Fourth, we control for additional control variables to mitigate endogeneity. Corruption in the host country, institutional distance, and geographical distance may affect the establishment mode (Doh et al., 2003; Arslan and Larimo, 2011; Rienda et al., 2021). We further control for the incorruptibility in the host country, the institutional distance, and the country-fixed effect. The country-fixed effect will control for the factors that changed with the host country but not with time, such as geographical distance. Column (4) of Table 7 shows the regression result after adding additional control variables. The coefficient of *SENT* is still significantly positive. However, the coefficients of the added control variables *INC* and *ID* are insignificant, while the coefficients of *MS* and *MG* are also insignificant. The reason may be that the country-level control variables are highly collinear with the country-fixed effect. Nevertheless, the coefficient of *SENT* that we are mainly concerned about remains unchanged.

After the above additional tests, we can believe that the main conclusion of this paper is robust.

## 7. Conclusion

We investigate the impact of investor sentiment on the establishment mode of OFDI. Specifically, we conduct an empirical study using the data of Chinese listed companies and use stock mispricing as the proxy for firm-level investor sentiment. The empirical results support our hypothesis that firms prefer cross-border acquisitions over greenfield investments when investor sentiment is high, and equity issuance and catering to sentiment are transmission channels; that is to say, high investor sentiment reduces the cost of equity financing and increases the scale of equity issuance, which is conducive to meeting the financial resources required by firms to carry out cross-border acquisitions, improving the ability of firms to carry out cross-border acquisitions. Moreover, when investor sentiment is high, cross-border acquisition meets investors’ pursuit of projects with high risk but long-term high-return for that cross-border acquisition is riskier than greenfield investment but is more conducive to firms’ rapid access to the foreign market and to firms’ acquiring advanced foreign technology and ready assets than greenfield investment is. Rational managers will cater to investors’ preferences, so they are more willing to carry out cross-border acquisitions rather than greenfield investments when investor sentiment is high. The results of cross-sectional tests show that the impact of investor sentiment on the establishment mode of OFDI is concentrated in non-SOEs, and is stronger in non-eastern province firms and in technology-intensive OFDI.

As far as we know, this paper is the first to analyze the impact of investor sentiment on the establishment mode of OFDI, which enriches the literature on international business and behavioral finance. Our evidence not only supports the “cheap financial capital hypothesis” proposed by Baker et al. (2008) but also proposes and verifies the “catering” channel. The “catering” channel is a meaningful finding because it points out the influence of managers’ self-interest motives on firms’ cross-border investments under the irrationality of investors, and it is a supplement to the existing literature on firms’ cross-border investment drivers. Taking Chinese listed companies as the sample, we also conducted additional cross-sectional tests from the perspectives of the state ownership, the location of firms, and the type of OFDI, which are very helpful for further understanding the internationalization decisions of firms in emerging economies.

Our research also has implications for policy formulation and corporate management in emerging economies. For emerging economies, realizing the reverse technology spillover effect is an essential motivation of OFDI, which is of great significance to the technological progress and output of the home country (Potterie and Lichtenberg, 2001; Yang et al., 2013). The cross-border acquisition has advantages over greenfield investment in realizing the reverse technology spillover effect; therefore, the relevant government departments of emerging economies should pay full attention to the role of the stock market in promoting OFDI, especially in promoting cross-border acquisition. Firms’ managers should also pay full attention to the firm’s market value to facilitate the best choice of establishment mode of OFDI. Of course, we should also see the dark side of overly optimistic investor sentiment, such as causing the stock price crash (Fu et al., 2021) and the decline of corporate investment efficiency (Polk and Sapienza, 2009; Campello and Graham, 2013). The key to good use of investor sentiment is to keep investor sentiment within a reasonable range. We should prevent stock prices from radical rising and falling and investor sentiment from being pessimistic. To achieve those goals, the governments of emerging economies should strengthen the construction of the regulatory institution of the stock market.

Although this paper makes some contributions to the existing literature, it has some limitations. First, our samples come from China, an emerging market. The conclusions drawn from these samples may not apply to developed countries. Second, our samples come from listed companies, ignoring the impact of investor sentiment on the establishment mode of OFDI of unlisted companies. It has been found in previous literature that investor sentiment also has a significant impact on the investment of unlisted companies (Badertscher et al., 2019). We are also interested in whether investor sentiment significantly impacts the establishment mode of OFDI of unlisted companies and how it affects them. If such influence exists, it will undoubtedly expand the scope of application of our conclusion. However, just as analyzed by Badertscher et al. (2019), the transmission mechanism of the impact of investor sentiment on the investment of listed companies is different from that of unlisted companies; it is difficult to be studied in a paper with limited length; we look forward to supplementing these studies in the future.

## Data availability statement

The datasets presented in this study can be found in online repositories. The names of the repository/repositories and accession number(s) can be found at: China Stock Market & Accounting Research (CSMAR) Database, websites of the Ministry of Commerce of China: https://www.gtarsc.com; http://www.mofcom.gov.cn.

## Author contributions

LD contributed to the conception and design of the study, organized the database and performed the statistical analysis, and wrote the first draft of the manuscript. JC supervised the research and wrote sections of the manuscript. All authors contributed to the article and approved the submitted version.

## Funding

This study is supported by the National Natural Science Foundation of China (grant number 71571074).

## Conflict of interest

The authors declare that the research was conducted in the absence of any commercial or financial relationships that could be construed as a potential conflict of interest.

## Publisher’s note

All claims expressed in this article are solely those of the authors and do not necessarily represent those of their affiliated organizations, or those of the publisher, the editors and the reviewers. Any product that may be evaluated in this article, or claim that may be made by its manufacturer, is not guaranteed or endorsed by the publisher.
